# Consumption of Lutein and Zeaxanthin and Its Relation to the Level of Macular Pigment Optical Density in Thai Subjects

**DOI:** 10.1155/2022/6321778

**Published:** 2022-04-15

**Authors:** Wipada Sae-Lao, Kansuda Wunjuntuk, Taweesak Techakriengkrai, Prapaisri P Sirichakwal

**Affiliations:** ^1^Faculty of Agriculture, Kasetsart University, Bangkok 10900, Thailand; ^2^Institute of Nutrition, Mahidol University, Nakhon Pathom 73170, Thailand

## Abstract

The aim of the study is to determine dietary lutein and zeaxanthin (*L*/*Z*) consumption and to evaluate its association with macular pigment optical density (MPOD) in Thai subjects. *Methods*. This study was a cross-sectional study. A total of 120 ophthalmologically healthy subjects aged between 40 and 72 years were recruited from Bangkok and the vicinity area. Demographic data were collected using a questionnaire, while a semiquantitative food frequency questionnaire assessed the *L*/*Z* intake. MPOD was determined using the reflectometry method (VISUCAM 500®, Carl Zeiss Meditec AG). Pearson's correlation coefficient analyzed the relationship between *L*/*Z* consumption and MPOD. *Results*. The mean age of the participants was 50.7 ± 7.5 years. The mean consumption of *L*/*Z* was 3.03 ± 2.65 mg per day. The mean MPOD was 0.102 ± 0.023 density units. Consumption of foods rich in *L*/*Z*, including ivy gourd (*r* = 0.217, *p* < 0.05), Chinese flowering cabbage (*r* = 0.194, *p* < 0.05), balsam pear (*r* = 0.193, *p* < 0.05), lettuce (*r* = 0.182, *p* < 0.05), sweet corn (*r* = 0.181, *p* < 0.05), and pumpkin (*r* = 0.181, *p* < 0.05), was positively associated with the mean optical density (mean MPOD). Consumption of green onion (*r* = 0.212, *p* < 0.05) was positively associated with the sum of optical densities (MPOD volume). In contrast, chilli pepper consumption showed a negative association with mean MPOD (*r* = −0.220, *p* < 0.05) and amaranth showed a negative association with MPOD volume (*r* = −0.283, *p* < 0.05). No association was found between total *L*/*Z* consumption and MPOD. *Conclusion*. *L*/*Z* consumption is low among Thais living in Bangkok and the vicinity area, which may not be sufficient to ensure eye health, and total *L*/*Z* consumption is not associated with MPOD.

## 1. Introduction

Lutein and zeaxanthin (*L*/*Z*) are two fat-soluble antioxidants that belong to the class of carotenoids, the xanthophylls. Together with their conversion isomer meso-zeaxanthin, they are the main components of the macular pigment (MP), a compound concentrated in the macular region of the retina and responsible for protecting central vision [1]. The function of MP is thought to be both passive and active. First, its peak absorption spectrum of 460 nm helps to protect the macula from the phototoxicity of blue light. Second, it protects the macula from photochemical damage by acting as a free radical scavenger, thus having an antioxidant effect [2]. Previous studies show that low levels of MP are a risk factor for age-related macular degeneration (AMD), the leading cause of blindness in the Western world [3]. There was also evidence to support the hypothesis that MP can protect against AMD [2].

Increased dietary *L*/*Z* intake increases serum concentrations and macular pigment optical density (MPOD). These markers are associated with improved visual function and a lower risk of AMD [4]. Many epidemiological studies and intervention trials have investigated carotenoid intake and levels using dietary questionnaires or by measuring *L*/*Z* plasma concentrations. Several studies found that *L*/*Z* consumption and plasma levels were inversely correlated with AMD risk [5, 6]. One of the first comprehensive studies on carotenoids is the Eye Disease Case-Control Study, which compared diet with the risk of developing AMD. The results showed that people with high levels of *L*/*Z* in their blood had a significantly lower risk of developing eye diseases. In addition, those who consumed diets that contained the most *L*/*Z* (up to 5.8 mg/day) had a significantly lower risk of AMD than those whose diets contained the least amount (up to 1.2 mg/day) [7]. A prospective cohort study with cohorts from the Nurses' Health Study and the Health Professionals Follow-up Study in the USA suggests that a higher intake of bioavailable *L*/*Z* is associated with a lower long-term risk of advanced AMD [8]. Similar results were found in a recent analysis of a national dietary survey, the Third National Health and Nutrition Examination Survey or NHANES III. This analysis also showed that consumption of 6 mg/day *L*/*Z* was associated with a lower risk of developing AMD [9].

In Thailand, the consumption of these two carotenoids has never been studied. However, there is a high prevalence of visual impairment in the Thai population. According to the 4th National Survey of Blindness, Low Vision, and Major Eye Diseases in Thailand in 2006, AMD was the third leading cause of blindness after cataracts and glaucoma. 799,296 Thais suffer from AMD, and 21,425 people are irreversibly blind due to AMD [10]. The incidence of AMD is expected to increase with the age of the population. Since the optimal consumption of *L*/*Z* for Thais is not known and the association between *L*/*Z* consumption and MPOD in Thais has not been researched, the present study aimed to determine *L*/*Z* consumption in Thais living in Bangkok and vicinity area and to investigate the association with MPOD.

## 2. Materials and Methods

### 2.1. Ethical Approval

The Research Ethics Committee of Kasetsart University has approved this study. It was conducted following the international guidelines for the protection of human research, the Declaration of Helsinki, the Belmont Report, the guideline of the Council for International Organizations of Medical Sciences (CIOMS), and the International Conference Harmonization in Good Clinical Practice. The approval number was COA63/036.

### 2.2. Study Design

This study is cross-sectional research that collected 120 subjects from July to November 2020. All subjects were informed about the study and signed an informed consent form. They were eye-healthy participants of both genders, aged 40 and 72 years, and living in Bangkok and the vicinity areas. Despite this study, the participants may not represent the Thai population; it is essential to know the consumption of *L*/*Z* in an urban context. Foreigners, citizens who had difficulty answering the survey, and people not from the central region of Thailand, for example, the northeast or south, were excluded. Participants with ocular diseases (AMD, cataract, glaucoma) or physical or mental disabilities were also excluded.

A semiquantitative food frequency questionnaire (semi-FFQ) was developed based on the semi-FFQ developed and validated by Kositwechsakul et al., which provided *L*/*Z* intake values to data collected using a food record [11]. The semi-FFQ was applied with a specific list of 44 high *L*/*Z* foods commonly consumed in the urban Thai population, including eggs (chicken and duck) as an animal source, 33 vegetables, 6 legumes, 2 maize/corn products, and one nut. Lutein and zeaxanthin levels were estimated using data from the USDA [12] and studies conducted by Tharasena and Lawan [13], Chandra-Hioe et al. [14], and Pasaporte et al. [15]. Three nutritionists reviewed the semi-FFQ before starting the collection of *L*/*Z* intake.

Subjects were made to undergo ophthalmic examination which included best-corrected visual acuity, tonometry, and slit lamp examination to ensure that there is no ocular pathology. Both eyes were dilated with 1% w/v tropicamide solution eye drops in each subject. After 30 minutes, both eyes were photographed with the VISUCAM 500® digital fundus camera (Carl Zeiss Meditec, Jena, Germany), and the ophthalmologist selected MPD mode and captured a 30-degree image in the same way as other fundus camera images. The Visucam 500 measured MPOD by capturing images with a special blue filter and using the single wavelength reflectometry method described by Delori et al. [16] and Schweitzer et al. [17]. It displays quantitative information using four parameters: the area in which macular pigment is detectable (MPOD area), the sum of optical densities (MPOD volume), the maximum optical density (max MPOD), and the mean optical density (mean MPOD).

### 2.3. Study Design

Demographic data (age, gender) and consumption of *L*/*Z* were analyzed using descriptive statistics (mean and standard deviation). Pearson's correlation coefficient analyzed the relationship between *L*/*Z* consumption and MPOD. The effect of egg consumption on MPOD was analyzed using ANOVA. The prediction of the dietary factor influencing MPOD was analyzed using analytical statistics (regression analysis). A 95% confidence interval was used for all tests and the significance was defined as *p* < 0.05.

## 3. Results

A total of 123 subjects were recruited for this study. Three subjects were excluded due to a reported ocular disease (cataract). After applying these criteria, 120 participants (240 eyes) were enrolled in the study. The subjects who were included in the study reported healthy eyes.

### 3.1. Participants' Sociodemographic Characteristics

The characteristics of the participants are shown (see Table 1). The mean age of the participants was 50.7 ± 7.5 years. Among all participants, 75% were women, 60% were married, 45% have an income of more than 50,000 Baht/month, 65.8% live in Bangkok, the metropolis of Thailand, and 34.2% live in the vicinity areas. In terms of education, 68.3% of the participants have a university degree.

### 3.2. Dietary Intake of Lutein and Zeaxanthin

After considering the dietary intake of *L*/*Z* among participants, the food that the participants chose to consume and contributed the highest dietary *L*/*Z* content were Chinese bitter gourd, Chinese kale, Chinese cabbage, morning glory, chicken egg, spinach, amaranth, sweet corn, pumpkin, and broccoli. The top ten foods with the highest *L*/*Z* content in the participants' diets are listed (see Table 2).

When considering the dietary intake of *L*/*Z* for a particular age group, gender, and residential area, we found that participants in the age group 50–59 years consumed 3.25 ± 3.03 mg *L*/*Z* per day, while the age group <50 and 60+ consumed 3.00 ± 2.58 mg/day and 2.58 ± 1.79 mg/day, respectively. However, there was no significant difference in the mean of *L*/*Z* intake among the age group. The female participants consumed 3.08 ± 2.54 mg/day *L*/*Z*, while the male participants consumed 2.89 ± 2.99 mg/day *L*/*Z*. There was no significant difference in comparison between the *L*/*Z* intake for male and female. Participants who lived in the vicinity areas consumed 3.77 ± 3.10 mg/day *L*/*Z*, while those in Bangkok consumed 2.70 ± 2.38 mg/day *L*/*Z*. However, we found no significant difference in *L*/*Z* intake for participants lived in Bangkok and vicinity areas. The lowest and highest values for dietary consumption of *L*/*Z* among participants were 0.2 mg/day and 11.98 mg/day, respectively. The mean dietary consumption of *L*/*Z* for all participants was 3.03 ± 2.65 mg/day. The mean, maximum, and minimum dietary intake of *L*/*Z* for each group/category are shown (see Table 3).

When participants were divided into tertiles based on dietary *L*/*Z* intake, half of the participants (50%) consumed more than 2 mg per day, 27.5% of the participants consumed 1-2 mg per day, while 22.5% of the participants consumed less than 1 mg per day. The number of participants in each tertile is described (see Table 4).

**Figure 1:**
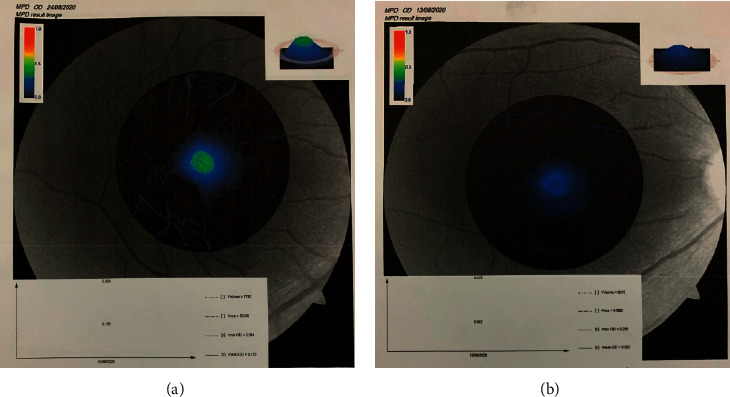
Comparison of the right eye of 2 participants with different MPOD values, analyzed by VISUCAM 500. (a) Participant with high MPOD, coded green-yellow (mean MPOD of the right eye = 0.155 density unit) and (b) participant with low MPOD, coded blue (mean MPOD of the right eye = 0.082 density unit).

**Figure 2:**
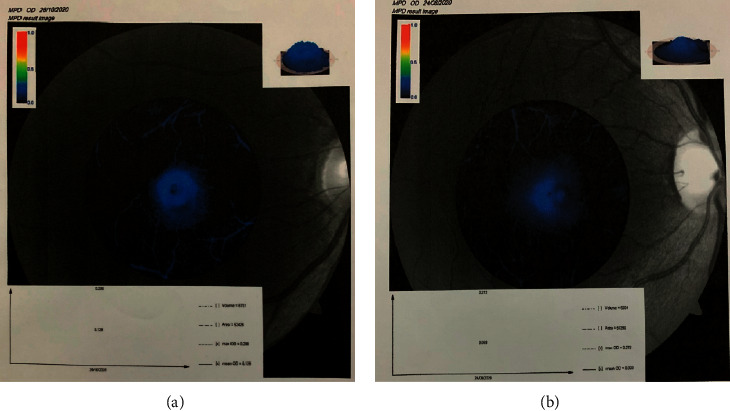
Comparison of MPOD image of 2 participants with different *L*/*Z* intakes. (a) Participant with an average of 11.98 mg *L*/*Z* per day (mean MPOD of the right eye = 0.128 density unit) and (b) participant with an average of 0.20 mg *L*/*Z* per day (mean MPOD of the right eye = 0.093 density unit).

The MPOD image captured by the VISUCAM 500 fundus camera from 2 participants with different MPOD values is presented (see Figure 1). A color-coded graph superimposed on the MPOD image, and a 3D map provided additional qualitative information about the MPOD in addition to the mean MPOD values. The participant with the green-yellow-coded 3D map showed a higher MPOD compared to the participant with the blue coding (Figure 1). The MPOD images for the participant with the highest intake of *L*/*Z* and the lowest intake of *L*/*Z* are shown (see Figure 2). The average MPOD of the participants is shown (see Table 5).

### 3.3. Association between *L*/*Z* Intake and MPOD

When correlating the amount of *L*/*Z* contributed by a *L*/*Z*-rich diet with the sum of optical densities (MPOD volume) and the mean optical density (mean MPOD), we found a significant positive association between consumption of six foods and mean MPOD, including ivy gourd (*r* = 0.217, *p* < 0.05), Chinese flowering cabbage (*r* = 0.194, *p* < 0.05), balsam pear (*r* = 0.193, *p* < 0.05), lettuce (*r* = 0.182, *p* < 0.05), sweet corn (*r* = 0.181, *p* < 0.05), and pumpkin (*r* = 0.181, *p* < 0.05), while consumption of chilli pepper showed a significant negative association with mean MPOD (*r* = −0.220, *p* < 0.05) (see Table 6). Furthermore, we found a significant positive association between intake of green onion and MPOD volume (*r* = 0.212, *p* < 0.05), while intake of amaranth showed a significant negative association with MPOD volume (*r* = −0.283, *p* < 0.05) (see Table 7).

The consumption of eggs is not associated with MPOD in this study. However, when focusing on participants with moderate egg consumption (≥3 eggs/week), moderate egg consumption affected MPOD, both within the same age group and between age groups. The mean MPOD for participants with moderate egg consumption (≥3 eggs/week) and low egg consumption (<3 eggs/week) in different age groups are presented (see Table 8). There was a significant effect of moderate egg consumption (≥3 eggs/week) on MPOD at the level of *p* < 0.001 for the three age groups (<50 age group, 50–59 age group and ≥60 age group) (*F* (2,117) = 51.023, *p*=0.000) (see Table 9). In this study, no association was found between total *L*/*Z* dietary intake and MPOD. When participants were divided into tertiles based on dietary *L*/*Z* intake and the predictor (dietary *L*/*Z*) of MPOD was determined, no significant difference in MPOD was found between tertiles (<1 mg *L*/*Z*/day, 1-2 mg *L*/*Z*/day, and >2 mg *L*/*Z*/day) in this study and dietary *L*/*Z* consumption was not found to be a predictor of MPOD.

## 4. Discussions

The results show that the Thai subjects had a low intake of *L*/*Z* (3.03 ± 2.65 mg per day). These results are consistent with a study conducted in the United States (US), which reported an intake of 1.7 mg of lutein per day [18]. The US Institute of Medicine also reported that American adults typically consume about 1-2 mg of lutein daily [19]. A study of healthy Spaniards aged 45–65 years also found an intake of 1.3 mg per day [20]. An Australian study of older adults living in the Blue Mountains found an even lower average intake of *L*/*Z*, reported at 0.9 mg/day [21]. Although several studies in Asia reported slightly higher intakes of *L*/*Z*, for example, a study of Chinese pregnant women in coastal regions with a healthy maternal fruit and vegetable intake, an average intake of *L*/*Z* of 3.3 ± 0.41 mg/day was reported [22]. In contrast, data from the Singapore Chinese Health Study reported a mean *L*/*Z* intake was 1.8 mg/day [23]. A study in the Indonesian population reported an average intake of *L*/*Z* of 1.1 mg/day [24]. These low levels of consumption may not be sufficient to promote eye health, especially in AMD prevention. There is currently a burgeoning interest in establishing Dietary Reference Intakes (DRI) guidelines for nonessential bioactive compounds such as lutein [25]. Establishing dietary guidelines for lutein for individual countries, especially Thailand, would promote the consumption of lutein-containing foods and raise public awareness of the potential health benefits of lutein.

It is worth noting that the foods that contributed significantly to *L*/*Z* consumption in a Thai sample were green leafy vegetables such as Chinese kale, Chinese cabbage, morning glory, spinach, and amaranth, while Chinese bitter gourd, which is commonly consumed in Thai households, contributed the most to *L*/*Z* consumption. Besides green vegetables, the most consumed *L*/*Z* food in this study population were egg, sweet corn, and pumpkin. It is precious to consider the bioavailability of phytochemicals in these foods, as they are mostly plant sources. A previous study has shown that fat and cooking method affect the absorption of carotenoids in these foods [26]. Egg yolk was the only food source of animal origin reported for *L*/*Z* in this study. It should be noted that other authors have described egg yolk as a good source of carotenoid content [27].

A previous study [28] reported the association of *L*/*Z* consumption with age and gender. However, in the present study, we found no significant difference in *L*/*Z* intake among age groups. In addition, we found no significant difference in comparison between the *L*/*Z* intake for male and female. Furthermore, we found no significant difference in *L*/*Z* intake for participants lived in Bangkok and vicinity areas. To understand demographic factors that influence eating habits, further studies that consider essential cultural and social aspects related to the eating behavior are needed.

Since humans cannot synthesize the macular pigment, *L*/*Z* are the main components, and they only obtain *L*/*Z* through the diet. Many studies [20, 29] have shown that diet, especially *L*/*Z* intake, influences macular pigment levels, which was also demonstrated in our study. We found a significant positive association between green and yellow vegetables, including ivy gourd, Chinese flowering cabbage, balsam pear, lettuce, sweet corn, and pumpkin, with the mean MPOD. Moreover, we also found that green onion showed a significant positive association with the sum of optical densities (MPOD volume). These findings are consistent with the study conducted in China, which reported that MPOD was positively associated with the consumption of Chinese wolfberries and green vegetables [30]. The study conducted in Spanish subjects reported that *L*/*Z* provided by red/orange foods and fruits had a stronger association with MPOD than green vegetables [31]. However, this study found a negative association between chilli pepper consumption and mean MPOD. Moreover, we also found a negative association between amaranth consumption and MPOD volume. Further studies should be conducted to determine the bioavailability of the Thai diet, especially spicy dishes, and the effect of cooking method on the absorption of *L*/*Z* in the Thai population.

A previous study revealed that daily consumption of egg has beneficial effects on MPOD [32]. In this study, no association was found between egg consumption and MPOD. However, when we focused on participants with moderate egg consumption (≥3 eggs/week), we found that moderate egg consumption appeared to affect MPOD when considered within the same age group and between age groups. Further studies should be conducted in the Thai population to better understand the effects of eggs on MPOD and the bioavailability of macular pigment in egg yolk in different age groups.

Many studies reported the association between *L*/*Z* intake and MPOD [33, 34]. However, in this study, no association was found between total consumption of *L*/*Z* and MPOD. This could be due to limitations of this study. Apart from dietary habit, MPOD also depends on several factors including age, gender, tobacco smoking, body mass index, and genetic background [35]. Those confounding factors should be considered. For the present study, despite factors that influence MPOD were analyzed by multiple regression, the total dietary intake of *L*/*Z* was not found to be a significant influencing factor for MPOD. The results are consistent with a recent study in the Mexican population, which only found a correlation between serum concentrations of *L*/*Z* and MPOD, but not with *L*/*Z* dietary intake [36]. Further studies to investigate serum concentration of *L*/*Z* and the bioavailability of *L*/*Z* in the Thai population should be conducted.

## 5. Conclusion

There is growing scientific evidence that MPOD can protect against AMD. Therefore, it is critical to understand the relationships between dietary intake, serum, retinal, and other tissue concentrations of *L*/*Z* and MPOD, especially in a specific population with different dietary habits and cultures. This study revealed data on vegetables and egg consumption among Thai subjects which reflected dietary status in Thailand. This study obtained statistically significant correlation between some vegetable intake and MPOD values. However, this study has 2 limitations. The first is the difficulty in collecting dietary data through a semi-FFQ. This method involves a self-report and affects reliability of data. Further study on serum *L*/*Z* should be performed. The second is the lack of previous research studies on MPOD and *L*/*Z* intake in the Thai population. To better understand the consumption *L*/*Z* and its correlation with MPOD, further studies in a wider Thai population are needed. This aim requires the collaboration of researchers with integrative interests, including nutrition, epidemiology, ophthalmology, biochemistry, and vision sciences.

## Figures and Tables

**Table 1:** Participant characteristics.^*∗*^

Variable	Unit/categories	Mean ± SD/*n* (%)
Age	Year old	50.7 ± 7.5

Gender	Female	90 (75)
	Male	30 (25)

Education	Below high school	15 (12.5)
	High school	23 (19.2)
	University	82 (68.3)

Marital status	Married	72 (60)
	Single	48 (40)

Income	<THB25,000/month	39 (32.5)
	THB25,000–50,000/month	27 (22.5)
	>THB 50,000/month	54 (45)

Residence	Bangkok	79 (65.8)
	Vicinity areas	41 (34.2)

^*∗*^Data presented as mean ± SD or frequency and percentage. *n* = Number of participants, THB = Thai Baht, Bangkok = the capital of Thailand, vicinity areas = Nakhon Pathom, Pathum Thani, Nonthaburi, Samut Prakan, and Samut Sakhon.

**Table 2:** Daily intake of *L*/*Z*^*∗*^ of the top ten foods.

Food lists	Scientific name	*L*/*Z* consumption mean ± SD (mg/day)
Chinese bitter gourd	*Momordica charantia*	0.94 ± 1.04
Chinese kale	*Brassica alboglabra*	0.46 ± 0.63
Chinese cabbage	*Brassica rapa*	0.39 ± 0.47
Morning glory	*Ipomoea aquatica*	0.35 ± 0.36
Chicken egg	*Gallus gallus*	0.29 ± 0.25
Spinach	*Spinacia oleracea*	0.51 ± 0.83
Amaranth	*Amaranthus viridis*	0.46 ± 0.76
Sweet corn	*Zea mays*	0.16 ± 0.24
Pumpkin	*Cucurbita moschata*	0.16 ± 0.20
Broccoli	*Brassica oleracea*	0.14 ± 0.21

^*∗*^Values are means ± SDs.

**Table 3:** Mean intake, maximum intake, and minimum intake of dietary *L*/*Z*^*∗*^ for each group of participants.

Variable	Unit/categories	Mean ± SD	Max	Min
Age	<50 years	3.00 ± 2.58	9.75	0.20
	50–59 years	3.25 ± 3.03	11.98	0.28
	≥60 years	2.58 ± 1.79	6.12	0.21

Gender	Female	3.08 ± 2.54	10.65	0.20
	Male	2.89 ± 2.99	11.98	0.27

Residence	Bangkok	2.70 ± 2.38	10.65	0.20
	Vicinity areas	3.77 ± 3.10	11.98	0.28

All participants	Total	3.03 ± 2.65	11.98	0.20

^*∗*^Unit = mg per day, max = maximum intake of *L*/*Z*, and min = minimum intake of *L*/*Z*.

**Table 4:** Number of participants in each tertile based on consumption of dietary *L*/*Z*.

Tertiles^*∗*^	Number of participants	%
<1 mg	27	22.5
1-2 mg	33	27.5
>2 mg	60	50.0
Total	120	100.0

^*∗*^Unit = average dietary consumption of *L*/*Z* per day (mg/day).

**Table 5:** Mean MPOD (volume, area, maximum, and mean). (*n* = 113, excluded subjects partially completed questionnaires).

	Mean ± SD
MPOD volume	6,197.49 ± 1,745.37
MPOD area	61,127.66 ± 12,771.37
Max MPOD	0.269 ± 0.052
Mean MPOD	0.102 ± 0.023

Values of maximum and mean MPOD in density units (d.u.); volume measured in d.u. degrees^2^; area measured in degrees^2^; MPOD: macular pigment optical density; max: maximum.

**Table 6:** Correlation between mean MPOD and the intake of 7 foods rich in *L*/*Z* among participants (*n* = 120).

	Ivy gourd	Chinese Flowering cabbage	Balsam pear	Lettuce	Sweet corn	Pumpkin	Chilli peppers
Mean MPOD	0.217^*∗*^	0.194^*∗*^	0.193^*∗*^	0.182^*∗*^	0.181^*∗*^	0.181^*∗*^	−0.220^*∗*^

^*∗*^Correlation is significant at the 0.05 level (2-tailed).

**Table 7:** Correlation between MPOD volume and the intake of green onions and amaranth among participants (*n* = 120).

	Green onions	Amaranth
MPOD volume	0.212^*∗*^	−0.283^*∗*^

^*∗*^Correlation is significant at the 0.05 level (2-tailed).

**Table 8:** Mean MPOD for the participant with low (<3 eggs/week) and moderate egg consumption (≥3 eggs/week) in the three age groups (<50 years, 50–59 years and ≥60 years).

Age group	Egg consumption	Mean MPOD ± SD	Number of participants
<50 years	<3 eggs/week	0.087 ± 0.015	16
	≥3 eggs/week	0.088 ± 0.018	46
	Total	0.087 ± 0.017	62

50–59 years	<3 eggs/week	0.120 ± 0.016	9
	≥3 eggs/week	0.110 ± 0.016	33
	Total	0.112 ± 0.016	42

≥60 years	<3 eggs/week	0.124 ± 0.019	7
	≥3 eggs/week	0.134 ± 0.018	9
	Total	0.129 ± 0.019	16

Values of mean MPOD in density units (d.u.); SD = standard deviation.

**Table 9:** ANOVA table shows the effect of moderate egg consumption (≥3 eggs/week) on mean MPOD.

			Sum of squares	df	Mean square	*F*	Sig.
Mean MPOD	Between groups	(Combined)	0.029	2	0.015	51.023	0.000
	Within groups		0.034	117	0.000		
	Total		0.063	119			

ANOVA analyzed at the *p* < 0.001 level for the three age groups (<50 age group, 50–59 age group and ≥60 age group).

## Data Availability

The dietary consumption of *L*/*Z* and MPOD data used to support the findings of this study are available from the corresponding author upon request.
